# A new species of the live-bearing fish genus *Poeciliopsis* from northern Mexico (Cyprinodontiformes, Poeciliidae)

**DOI:** 10.3897/zookeys.883.37586

**Published:** 2019-10-28

**Authors:** Kevin W. Conway, Mariana Mateos, Robert C. Vrijenhoek

**Affiliations:** 1 Department of Wildlife and Fisheries Sciences, Texas A&M University, College Station, TX 77843, USA; 2 Biodiversity Research and Teaching Collections, Texas A&M University, College Station, TX 77843, USA; 3 Monterey Bay Aquarium Research Institute, 7700 Sandholdt Road, Moss Landing, CA 95039, USA

**Keywords:** Taxonomy, Poeciliinae, Sonoran Desert, unisexual reproduction, gill rakers

## Abstract

*Poeciliopsis jackschultzi***sp. nov.**, is described based on seven specimens (17.9–26.7 mm SL) from the Río Concepción (also known as Río Magdalena), Sonora, Mexico. The new species belongs to the *Leptorhaphis* species group and can be distinguished from other members of this group by features of the skeleton and colouration. The new species is sympatric with *P. occidentalis*, a hybridogenetic all-female biotype *P. monacha-occidentalis*, and hybrids between *P. monacha-occidentalis* females and *P. jackschultzi* males. The distribution of *P. jackschultzi* is highly restricted, and the main habitat, spring-fed marshy streams and pools, is susceptible to loss and degradation in a desert environment with increasing human water demand.

## Introduction

The live-bearing fish genus *Poeciliopsis* (subfamily Poeciliinae, tribe Girardinini Lucinda & Reis, 2005) is distributed from southern Arizona (USA) southwards through western Central America to Colombia (Rosen and Bailey 1963: map 17). *Poeciliopsis*is among the few vertebrate taxa known to include all-female hybrid forms that reproduce asexually via gynogenesis or hybridogenesis, and has served as a model system for studying the evolution of unisexual reproduction (Schultz 1969; Vrijenhoek et al. 1989; Avise 2015). The genus was last revised by Rosen and Bailey (1963) who provided a rediagnosis based largely on osteological characters and divided the known species between two subgenera, *Poeciliopsis* and *Aulophallus*. The genus currently contains 24 valid species, 21 of which are classified in subgenus Poeciliopsis and three in subgenus Aulophallus (Mateos et al. 2002, 2019).

Prior to Rosen and Bailey (1963), Miller (1960) classified six species of *Poeciliopsis* into what he referred to as the *Leptorhaphis* species group (based on the generic name *Leptorhaphis* Regan, 1913; type species *Gambusia infans* Woolman, 1894), characterised by the presence of a jet black male colouration during courtship, the presence of a small, retrorse hook at the tip of the gonopodium, and the arrangement of the oral jaw teeth into an outer and inner row on the dentary and premaxilla. Miller (1960) originally classified *P. infans*, *P. porosus* (= *P. infans*), *P. lucida*, *P. occidentalis* s. s. and *P. sonoriensis* within this species group. *Poeciliopsis prolifica* was added subsequently to this species group by Mateos et al. (2002) based on the results of a molecular phylogenetic investigation of mitochondrial DNA sequences. A more recent molecular phylogenetic investigation of *Poeciliopsis* (Mateos et al. 2019) based on mitochondrial and nuclear DNA sequences recovered a monophyletic *Leptorhaphis* species group comprising four named species (*P. infans*, *P. lucida*, *P. occidentalis* s. l., and *P. prolifica*) and one undescribed species from the Río Concepción (Sonora, Mexico) (see below). Relationships among the members of the *Leptorhaphis* species group were largely unresolved in this multi-locus topology, but the grouping was firmly nested within the “predominantly Northern” clade of the subgenus Poeciliopsis, which also included *P. balsas*, *P. monacha* and *P. viriosa*.

In the early 1980s, one of us (RCV) collected individuals of *Poeciliopsis* at marshy localities in the Río Concepción drainage about 24 km south of Nogales (Sonora State). In addition to locally abundant *P. occidentalis* s. s. and the sperm-dependent, all-female, hybridogenetic biotype *P. monacha*-*occidentalis*, the samples included some unusual individuals that differed in colouration from other described members of the *Leptorhaphis* species group. Male and female specimens of this unrecognised taxon were reared in the laboratory and found to reproduce sexually. Subsequent multi-locus allozyme studies revealed that the unknown sexual species had several unique alleles, including a diagnostic “fast” allele at the *Pgd* locus. Except for some novel alleles, the new species was first interpreted to be a mosaic composed of genes derived from *P. occidentalis* and the hemiclonal *monacha* genome from hybridogenetic *P. monacha-occidentalis* (see Schenk 1992; Vrijenhoek 1993), but this initial inference was later shown to be incorrect. The new species is unique based on a recent analysis of nuclear and mitochondrial DNA sequences that place it near the root of the *Leptorhaphis* species group (Mateos et al. 2019). Retention of shared ancestral allozyme polymorphisms in this species likely explains the genomic mosaicism. Nonetheless, introgression from *P. monacha*-*occidentalis* hemiclones or *P. occidentalis* into the new species gene pool has not been ruled out and awaits evaluation at a broader genomic level. The purpose of this study is to provide a formal description for this undescribed species of *Poeciliopsis* from the Río Concepción.

## Materials and methods

Specimens of the new species were collected between 1999 and 2001. They were identified on the basis of one or both of the following traits: (a) homozygosity for the diagnostic “fast” *Pgd* allele (see Schenk 1992), following the methods described in Mateos and Vrijenhoek (2004); and (b) a mitochondrial gene sequence (e.g., Cytochrome b) that is distinct from all other *Poeciliopsis* (Mateos et al. 2019). The PGD protein (6-phosphogluconate dehydrogenase or 6Pgdh) is expressed in numerous tissues including fins, which enables “non-destructive” and rapid (~ 3 h) genotyping of specimens from a caudal fin clip that can regrow, or from a small clip of a pectoral fin from frozen specimens. Other specimens were obtained from museum collections with the following abbreviations:

**CNP-IBUNAM**Colección Nacional de Peces, Instituto de Biología, Universidad Nacional Autónoma de México, Mexico City;

**TCWC**Biodiversity Research and Teaching Collection, Texas A&M University, College Station;

**UMMZ**University of Michigan Museum of Zoology, Ann Arbor.

Counts and measurements generally follow Bussing (2008). Measurements were taken point to point to the nearest 0.1 mm using digital callipers. The number in parentheses following a meristic value denotes the frequency of that value. An asterisk denotes the value obtained from the holotype (if available). Select specimens were cleared and double stained for bone and cartilage following the protocol of Taylor and Van Dyke (1985) or cleared and single stained for bone only by omitting the cartilage staining step in the protocol of Taylor and Van Dyke (1985). Values reported for teeth, gill rakers, fin rays and vertebrae were obtained from cleared and stained specimens only. Alcohol-preserved specimens, cleared and stained specimens, or parts thereof were observed and photographed using a Zeiss SteReo Discovery V20 Microscope equipped with a Zeiss AxioCam MRc5 digital camera. Computed tomography (CT) scans of select specimens were also obtained at the Karel F. Liem BioImaging Center (Friday Harbor Laboratories, University of Washington) using a Bruker (Billerica, MA) SkyScan 1173 scanner with a 1 mm aluminium filter at 60 kV and 110 μA on a 2240 × 2240 pixel CCD at a resolution of 8.8 μm. Specimens were scanned simultaneously in a 50ml plastic Falcon tube (Corning, NY), in which they were wrapped with cheesecloth moistened with ethanol (70%) to prevent movement during scanning. The resulting CT data were visualised, segmented, and rendered in Horos (http://www.horosproject.org) and Amira (FEI).

General osteological terminology follows Rosen and Bailey (1963) and Parenti (1981). Gonopodial terminology follows Lucinda and Reis (2005). Canal neuromast terminology follows Tarby and Webb (2003) and Webb and Shirey (2003). The gill rakers of *Poeciliopsis* are morphologically heterogeneous. We refer to the different types using the following terminology: type 1a, an elongate, blade-like gill raker present only on the anterior edge of the first gill arch (typically in association with hypobranchial and ceratobranchial elements only); type 1b, as for type 1a but smaller, typically ¼ to ½ length of type 1a, may be present on posterior edge of gill arches 1–3 and anterior edge of arches 2–4 (typically in association with hypobranchial and ceratobranchial elements only); type 2, dorso-ventrally compressed gill rakers with 4 or 5 comb-like projections dorsally, forming a series of low parallel ridges along posterior edge of gill arch 4 (ceratobranchial only) and anterior edge of gill arch 5 (ceratobranchial 5); and type 3, a trifid gill raker with a central shaft similar in size and shape to the type 1b gill raker combined with a pair of lateral processes that extend from the base of the central shaft and support multiple minute conical teeth, present on the anterior edge of arches 2–4 (ceratobranchial only). Names of subgroups and species of *Poeciliopsis* follow Mateos et al. (2019).

Genetic distances (uncorrected p) reported were obtained using Paup*4.0a (build 165) (Swofford 2002) and derived from sequences available from Mateos et al. (2019).

## Taxonomy

### Poeciliopsis jackschultzi sp. nov.

Taxon classificationAnimaliaCyprinodontiformesPoeciliidae

83ADC3B6-D0BA-5671-B348-191CF7E1F079

http://zoobank.org/3949FE67-BD75-407B-8A23-2F41DD22F70E

Figures 1 , 2 , 3 , 4a , 5 , 6 , 7a , 8a , 9 Common name: Río Concepción topminnow (English); guatopote del Concepción (Spanish).

#### Holotype.

CNP-IBUNAM 23406, male, 20.3 mm SL; Mexico, Sonora, La Atascosa, tributary of the Alisos-Bambuto branch of the Río Concepción at highway 15 road crossing close to Rancho Semarnap, small spring at right side bank, 30°58'47.86"N, 110°52'21.07"W, M. Mateos and R. C. Vrijenhoek, 17 January 2001.

#### Paratypes.

TCWC 20082.01, 2 (C&S), 1 male/1 female, 19.0–23.0 mm SL; TCWC 20082.02, 2, 1 male/1 female, 17.9–26.7 mm SL; same data as holotype. – TCWC 20083.01, 1 (C&S), male, 20.2 mm SL; Mexico, Sonora, Rancho Las Playas, tributary of the Alisos-Bambuto branch of the Río Concepción, near town of La Providencia, 30°55'9.34"N, 110°51'38.25"W, M. Mateos, R. C. Vrijenhoek and L.A. Hurtado, 20 April 1999. – TCWC 20084.01, 1, male, 19.0 mm SL (DNA voucher); Mexico, Sonora, Cocospera-Babasac branch of the Río Concepción, at town of Imuris under Highway 15 bridge, 30°46'29.46"N, 110°51'28.80"W, M. Mateos, and R. C. Vrijenhoek, 11 May 2000.

#### Diagnosis.

A member of the *Leptorhaphis* species group (Miller 1960) based on the presence of a small, retrorse hook at the tip of the gonopodium and arrangement of the oral jaw teeth into an outer and inner row on the dentary and premaxilla. The new species can be distinguished from all other members of the *Leptorhaphis* species group and all other members of *Poeciliopsis* (except *P. monacha*) by the presence of type 3 gill rakers on the anterior edge of ceratobranchials 2–4. It is further distinguished from members of the *Leptorhaphis* species group by the following combination of characters: inner row of dentary and premaxilla with 7–10 weakly tricuspid teeth; 6 or 7 pores in preopercular portion of preoperculo-mandibular lateral line canal; a single ossification (posterior sclerotic) in scleral cartilage; a broken horizontal line comprising 15–17 small, dark-brown spots extending along posterior two-thirds of body; and the absence of a black spot at base of the anterior part of the dorsal fin.

#### Description.

**Figure 1.**
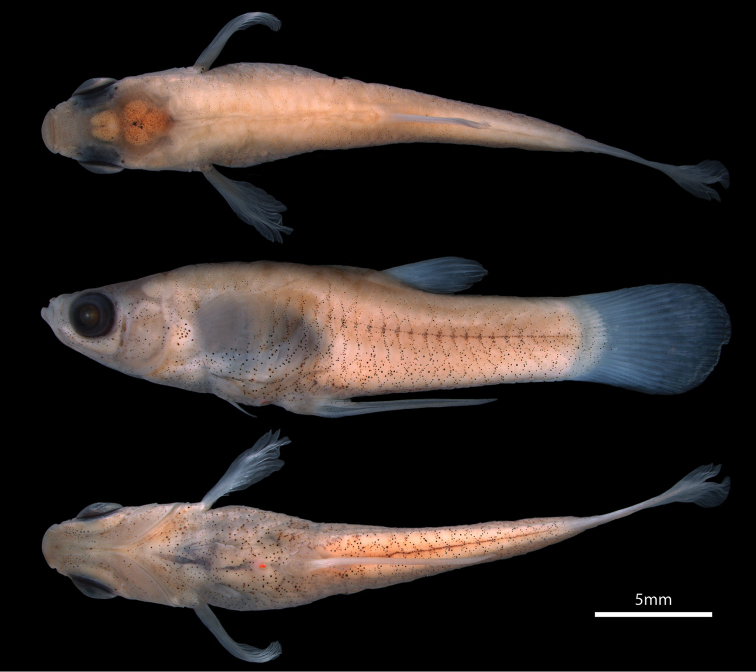
*Poeciliopsis jackschultzi*, CNP-IBUNAM 23406, holotype, male, 20.3 mm SL; Mexico, Sonora, Río Concepción.

**Figure 2.**
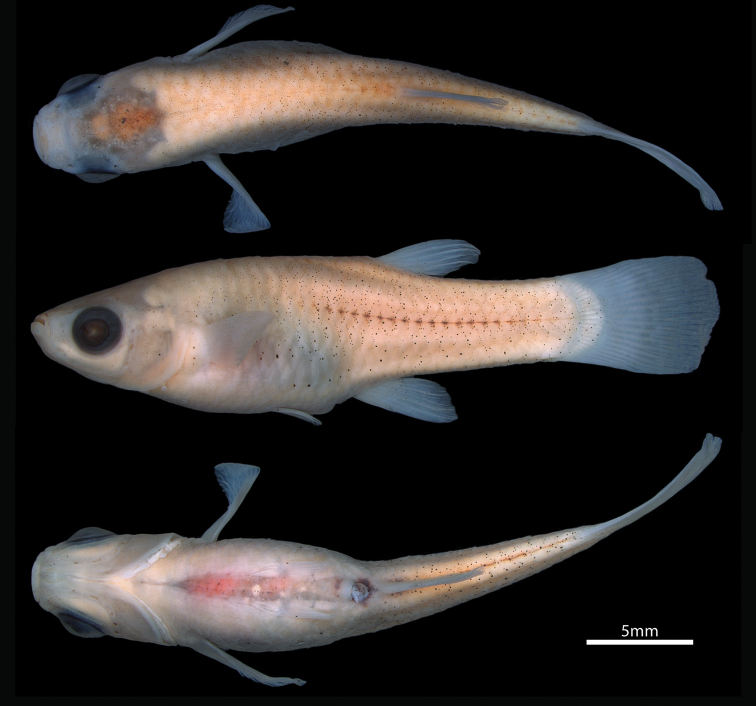
*Poeciliopsis jackschultzi*, TCWC 20082.01, paratype, female, 26.7 mm SL; Mexico, Sonora, Río Concepción.

Male and female body shapes as in Figures 1 and 2. Morphometric characters in Table 1. Predorsal and preanal profile convex; postdorsal profile slightly concave; postanal profile almost straight, from insertion of posteriormost anal-fin ray to caudal-fin base. Anterior half of body moderately compressed in male, round in female; posterior half of body compressed in both sexes. Body depth greatest at imaginary vertical line through origin of anal fin in male; at imaginary vertical line through vent in female.

**Table 1.** Select morphometric characters obtained from the holotype (male) and paratypes (2 males and 2 females) of *Poeciliopsis jackschultzi*. Ranges for males and females are separated (ranges reported for males include value obtained from holotype).

	**Holotype**	**Males (*N* = 3)**	**Females (*N* = 2)**
Standard length (SL)	20.3	17.9–20.3	23.0–26.7
Head length	27.1	24.5–29.0	25.6–29.6
Head width	16.3	16.2–16.8	17.8–18.3
Interorbital distance	10.8	10.0–10.9	9.6–11.9
Postorbital distance	12.8	10.7–12.9	10.9–12.7
Orbit length	7.9	7.9–9.2	7.8–9.5
Snout length	4.9	4.9–5.5	6.0–6.5
Body depth	22.2	21.4–22.7	19.6–21.3
Caudal peduncle depth	14.8	14.8–16.2	15.2–15.3
Predorsal distance	61.6	58.9–62.2	63.5–64.5
Preanal distance	49.3	45.8–50.0	58.4–60.0
Dorsal-fin origin to caudal-fin base	38.4	38.4–39.3	36.5–38.9
Anal-fin origin to caudal-fin base	54.7	47.0–55.6	40.8–42.7
Dorsal-fin length	18.7	17.8–21.8	16.9–19.8
Anal-fin length	36	35.9–38.8	16.9–17.9
Pectoral-fin length	19.7	19.4–19.8	16.9–19.8
Pelvic-fin length	8.4	5.4–8.4	8.2–8.7

**Figure 3.**
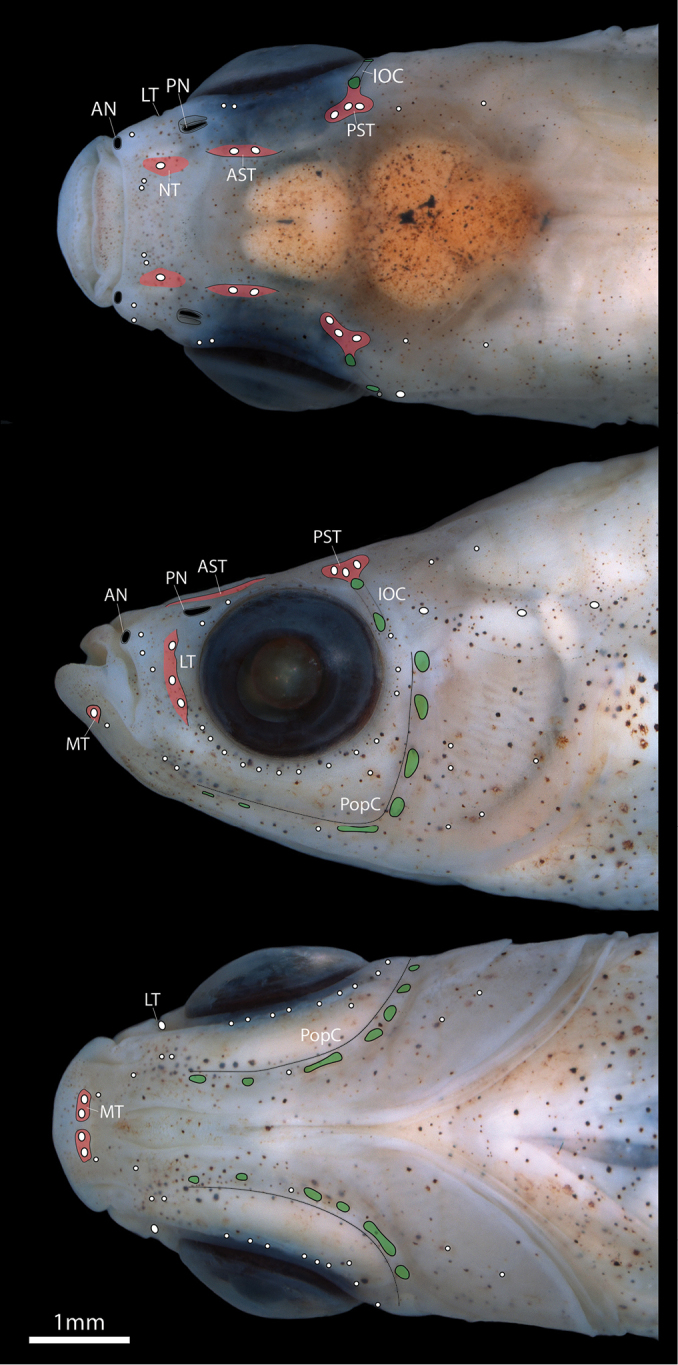
Head of *Poeciliopsis jackschultzi* in dorsal, lateral and ventral view (CNP-IBUNAM 23406, holotype, male, 20.3 mm SL). Sensory troughs containing presumptive canal neuromasts outlined in red. Pores in cephalic lateral line sensory canals in green. Small white circles represent superficial neuromasts. White ovals represent presumptive canal neuromasts. Abbreviations: AN, anterior nostril; AST, anterior supraorbital trough; IOC, infraorbital canal; LT, lachrymal trough; MT, mandibular trough; NT, nasal trough; PN, posterior nostril; PopC, preopercular canal; PST, posterior supraorbital trough.

**Figure 4.**
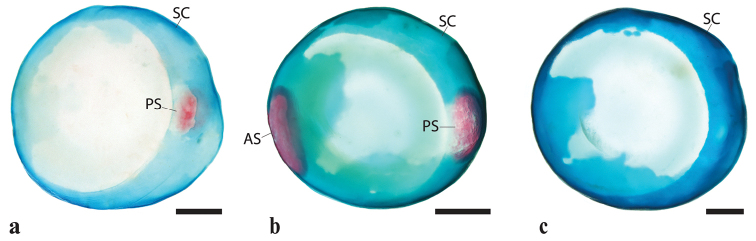
Sclerotic cartilage and sclerotic ossifications in members of *Poeciliopsis* (right side, image reversed) **a***P. jackschultzi*, TCWC 20082.01, paratype, female, 23.0 mm SL **b***P. lucida*, UMMZ 189041, female, 24.0 mm **c***P. occidentalis*, UMMZ 202393, female, 35.0 mm SL. Abbreviations: AS, anterior sclerotic. PS, posterior sclerotic; SC, scleral cartilage; Scale bar: 0.4 mm.

**Figure 5.**
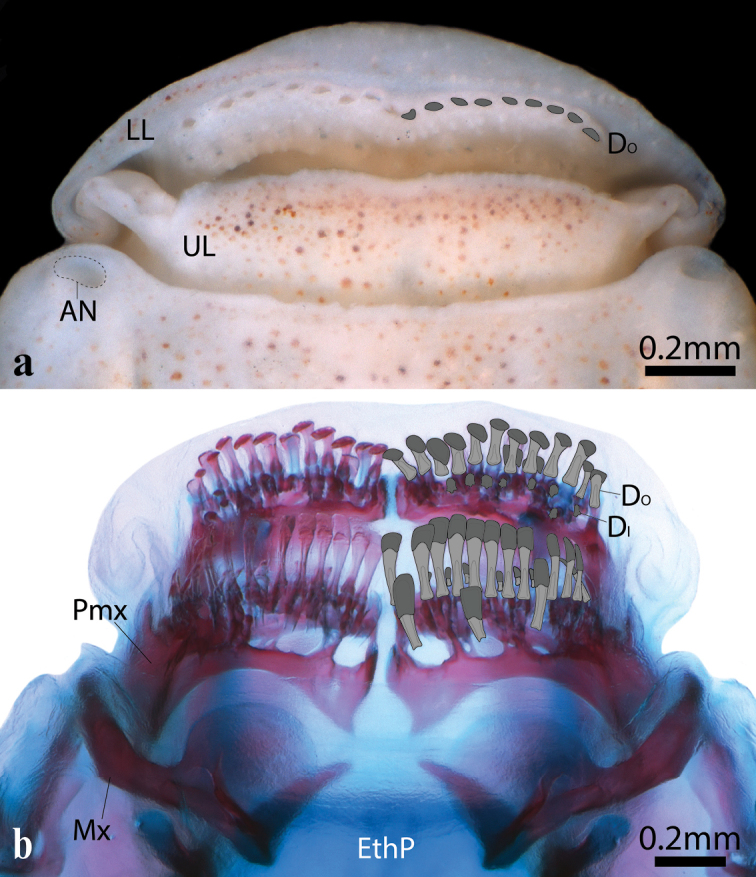
Mouth of *Poeciliopsis jackschultzi* in dorsal view (anterior to top of page) **a**CNP-IBUNAM 23406, holotype, male, 20.3 mm SL **b**TCWC 20082.01, paratype, female, 23.0 mm SL. Cusps of dentary teeth highlighted in dark grey on right side in a and b. Shaft of dentary teeth highlighted in light grey on right side in b. Abbreviations: AN, anterior nostril; D, dentary; DO, dentary teeth of outer row; DI, dentary teeth of inner row; EthP, ethmoid plate; Mx, maxilla; Pmx, premaxilla; LL, lower lip; UL, upper lip.

**Figure 6.**
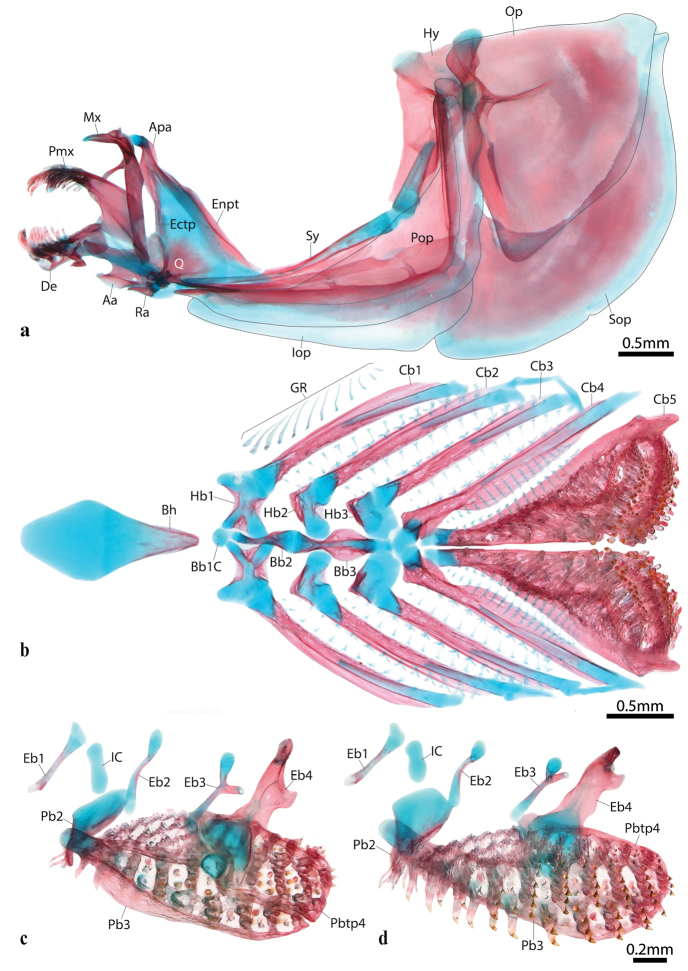
Viscerocranium of *Poeciliopsis jackschultzi* (TCWC 20082.01, paratype, female, 23.0 mm SL) **a** hyopalatine arch right side in lateral view (image reversed) **b** ventral gill arches in dorsal view **c** dorsal gill arches (right side) in dorsal view **d** dorsal gill arches (right side) in ventral view (image reversed). Opercular bones outlined in black in a. Scale bar shared between c and d. Abbreviations: Aa, anguloarticular; Apa, autopalatine; Bb1C, basibranchial 1 cartilage; Bb2-3, basibranchials 2–3; Bh, basihyal; Cb1-5, ceratobranchials 1–5; De, dentary; Eb1-4, epibranchial 1–4; Ectp, ectopterygoid; Enpt, endopterygoid; Hb1-3, hypobranchials 1–3; Hy, hyomandibular; IC, interarcular cartilage; Iop, interopercle; GR, gill raker; Mx, maxilla; Op, opercle; Pb2-3, pharyngobranchial 2–3; Pbtp4, pharyngobranchial toothplate 4; Pmx, premaxilla; Pop, preopercle; Q, quadrate; Ra, retroarticular; Sop, subopercle; Sy, symplectic.

Head and eye large. Anterior nostril a circular opening located at anterior tip of snout, lateral to upper lip (Fig. 3). Posterior nostril an oval opening, located medial to anterodorsal margin of orbit; a delicate fold of skin located lateral to posterior nostril. Single sclerotic bone (posterior) present in scleral cartilage (Fig. 4a). Mouth superior, almost aligned with upper margin of orbit. Two rows of teeth on premaxilla and dentary (Fig. 5). Teeth in outer row with spatula-shaped cusp, 2 or 3 times larger than teeth of inner row; teeth of inner row with weakly trifid cusp. Premaxilla with 10–12 teeth in outer row; 8–10 in inner row. Dentary with 9 or 10 teeth in outer row; 7–9 teeth in inner row. Upper pharyngeal jaw comprising teeth associated with ventral surface of pharyngobranchial 2, pharyngobranchial 3 and pharyngobranchial 4 toothplate (Fig. 6c, d). Teeth on pharyngobranchial 2 narrow, spatula-like, with small flattened cusp and elongate shaft; arranged randomly along ventral surface of bone. Teeth along anteromedial edge of ventral surface of pharyngobranchial 3 similar in size and shape to teeth on ventral surface of pharyngobranchial 2; arranged in three staggered rows (orientated along rostral-caudal body axis); each row comprising 8–9 or cohorts of 5–7 teeth arranged in a single row (orientated along medial-lateral body axis); each cohort located anterior to a deep crypt associated with development of replacement teeth. Teeth over remainder of ventral surface of pharyngobranchial 3 and pharyngbranchial 4 toothplate conical, with slightly recurved tip. Configuration of conical teeth similar to spatula-like teeth; arranged in multiple staggered rows each comprising multiple cohorts; each cohort comprising 4 or 5 teeth arranged in a single row (orientated along medial-lateral body axis) and located anterior to a deep replacement tooth crypt; size of tooth within each cohort increases gradually in a medial to lateral direction; largest conical teeth of upper pharyngeal jaw located along lateral edge of pharyngobranchial 3 and pharyngobranchial 4 toothplate. Lower pharyngeal jaw comprising teeth associated with dorsal surface of ceratobranchial 5 (Fig. 6b). Medial edge of ceratobranchial 5 with 4 or 5 widely spaced conical teeth, arranged in a single row (orientated along the rostral-caudal body axis). Remainder of teeth spatula-like, similar in shape to teeth located on ventral surface of pharyngobranchial 2; size of teeth increases gradually from anterior to posterior, with largest teeth located along posterior edge of bone. Arrangement of spatula-like teeth becoming more regular towards posterior, culminating in two dense bands of teeth each comprising 2 or 3 rows of ca. 13–15 teeth; a deep crypt associated with development of replacement teeth located anterior to each band of teeth.

**Figure 7.**
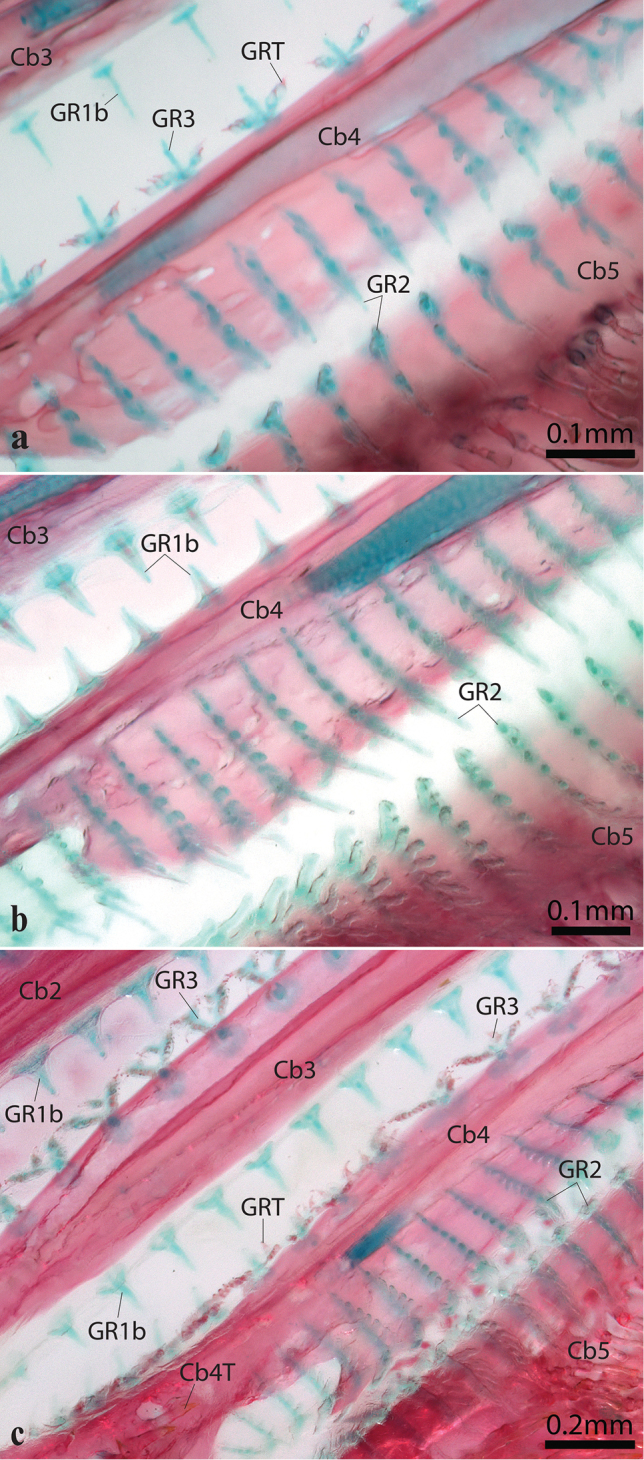
Gill rakers on ceratobranchials 2/3–5 in *Poeciliopsis***a***P. jackschultzi*, TCWC 20082.01, paratype, female, 23.0 mm SL **b***P. lucida*, UMMZ 189041, female, 24.0 mm SL **c***P. monacha*, UMMZ 178246, female, 22.0 mm SL. Gill filaments removed. Abbreviations: C2–5, ceratobranchials 2–5; Cb4T, teeth on ceratobranchial 4; GR1b, 2, 3, type 1b, 2, or 3 gill rakers; GRT, gill-raker tooth.

Gill rakers present on anterior and posterior margins of gill arches 1–4 and anterior margin of ceratobranchial 5 (Fig. 6b); number of gill rakers associated with each arch listed in Table 2. Anterior edge of gill arch 1 with 11 or 12 type 1a gill rakers associated with hypobranchial and ceratobranchial; posterior edge of gill arch 1–3 with 12–13 type 1b gill rakers associated with ceratobranchial. Anterior edge of gill arch 2–3 with 12–14 gill rakers, including 2 or 3 type 1b gill rakers associated with hypobranchial and lower part of ceratobranchial and 10 or 11 type 3 gill rakers associated with ceratobranchial (Fig. 7a). Anterior edge of gill arch 4 with 12–14 gill rakers along ceratobranchial, including 2 or 3 type 1b gill rakers and 10 type 3 gill rakers. Posterior edge of ceratobranchial 4 and anterior edge of ceratobranchial 5 with 16 or 17 type 2 gill rakers (Fig. 7a). Posterior edge of ceratobranchial 4 expanded as a flat membranous shelf to which base of gill rakers articulate (Fig. 7a).

**Table 2.** Counts of gill rakers in members of the *Leptorhaphis* species group and *P. monacha*. Gill raker type in parentheses (see materials and methods for details).

**Species**	**N**	**Gill arch 1**		**Gill arch 2**		**Gill arch 3**		**Gill arch 4**		**Gill arch 5**
		**Anterior**	**Posterior**	**Anterior**	**Posterior**	**Anterior**	**Posterior**	**Anterior**	**Posterior**	**Anterior**
*P. jackschultzi*	3	11–12 (1a)	12–13 (1b)	2–3 (1b)/10–11 (3)	13–14 (1b)	2–3 (1b)/10–11 (3)	14 (1b)	2–3 (1b)/10 (3)	17 (2)	16 (2)
*P. infans*	4	14–15 (1a)	18–20 (1b)	16–17 (1b)	18–19 (1b)	18–19 (1b)	18–20 (1b)	20–22 (1b)	18–19 (2)	18–19 (2)
*P. lucida*	4	14–15 (1a)	20–21 (1b)	20–21 (1b)	20–21(1b)	20–21 (1b)	19–21 (1b)	18–19 (1b)	20–21 (2)	19–21 (2)
*P. occidentalis*	4	11–12 (1a)	18–19 (1b)	19-20 (1b)	19–21 (1b)	18–20 (1b)	17–19 (1b)	18–19 (1b)	24–25 (2)	23–24 (2)
*P. prolifica*	4	10–11 (1a)	14–15 (1b)	14–16(1b)	14–16 (1b)	14–16 (1b)	14–16 (1b)	15–16 (1b)	18–20 (2)	15–16 (2)
*P. monacha*	4	12–13 (1a)	14–16 (1b)	13–14 (3)	15–16 (1b)	16–18 (3)	15 (1b)	15 (3)	17–18 (2)	16–17 (2)

Cephalic lateral line canal system with following components (Fig. 3): single large canal neuromast (stage IIa canal neuromast of Tarby and Webb 2003) located in shallow nasal trough (equivalent to nasal portion of supraorbital lateral line canal); two large canal neuromasts (stage IIa) located in shallow anterior supraorbital trough (anterior portion of supraorbital lateral line canal) dorsomedial to anterodorsal margin of orbit; three large canal neuromasts (stage IIa) located in shallow boomerang-shaped posterior supraorbital trough (posterior portion of supraorbital lateral line canal) dorsomedial to posterodorsal margin of orbit; three large canal neuromasts (stage IIb) located in dorso-ventrally elongate lachrymal trough anterior to orbit (lachrymal portion of infraorbital lateral line canal), edges of trough supported by flanges of bone extending from surface of lachrymal; two pores on dorsolateral surface of head posterior to orbit associated with dermosphenotic portion of infraorbital canal; two large canal neuromasts (stage IIa) located in shallow mandibular trough (mandibular portion of preoperculo-mandibular lateral line canal on dentary) located on lower jaw posterolateral to jaw symphysis; 6 or 7 pores of variable size associated with preopercular portion of preoperculo-mandibular lateral line canal. Lateral line canal on dermosphenotic and preopercle (Fig. 6a) an open trough of bone roofed by skin only (canal neuromasts inside of each canal stage III of Tarby and Webb 2003). Multiple superficial neuromasts placed over surface of head, most obvious on surface of skin bordering ventral margin of orbit (Fig. 3).

Dorsal-fin rays 8 (ii,4,ii or i,5,ii); anal-fin rays 10 (iii,6,i); pectoral-fin rays 14 (ii,9,iii); pelvic-fin rays 6 (i,3,ii or i,4,i). Total number of caudal-fin rays 29, comprising 15 (2) or 17 (1) principal rays, 13 (2) or 15 (1) branched rays. Dorsal procurrent rays 6, ventral procurrent rays 6 (1) or 7 (2). Total number of vertebrae 31, comprising 13 abdominal+18 caudal vertebrae. Ribs 11 or 12; epicentrals 9. First dorsal-fin pterygiophore inserting into interneural space between vertebrae 13/14 in both sexes. First anal-fin pterygiophore inserting into interhemal space between vertebrae 13/14 in female (not obtainable in males). 28 (3*) or 29 (1) scales in lateral series plus 1(1) or 2 (3*) scales on base of caudal fin; 16 (2) or 17 (1) predorsal scales (count not obtained from holotype); 16 scales around caudal peduncle.

**Figure 8.**
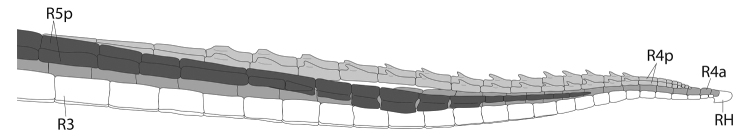
Schematic diagram of the distal tip of the gonopodium in *Poeciliopsis jackschultzi* (TCWC 20082.01, paratype, male, 19.0 mm SL). Abbreviations: R3, ray 3; R4a, ray 4 anterior branch (intermediate grey); R4p, ray 4 posterior branch (light grey); R5p, ray 5 posterior branch (dark grey).

Gonopodial complex composed of three functional gonoapophyses (modified hemal spines) and seven gonactinosts (modified proximal-middle pterygiophores). Second gonactinost a compound element; product of ontogenetic fusion of three proximal-middle pterygiophores. Ligostyle present. Gonopodium asymmetrical, sinistral (Fig. 8a). R3, unbranched with ~ 48 segments. Segments becoming progressively narrower distally; segment 1 largest element; distalmost segments tiny elements, approximately ¼ width of more proximal elements. Posterior margin of R3 with pronounced groove proximally (corresponding to segments 6–18), accommodating R4. R4, branched, branching point obscured by R3, segments of each branch difficult to count with precision. R4a without further division; extending to tip of gonopodium. R4p divided again at ca. midpoint along length; segments close to distal tip of sub-branches of R4p bearing a serra, forming a serration of ca.14 paired serrae along dorsal edge of gonopodium (Fig. 8). R5 branched; branching point ca. 11 segments distal to ray base; sub-branches R5a and R5p remain in close contact towards distal tip, displaced to left side of gonopodium, terminating between R3 and sub-branches of R4p proximal to distal tip of gonopodium. Small retrorse hook present at distal tip of gonopodium; confluent with distalmost segment of R3 and R4A.

#### Colouration.

In alcohol (Figs 1, 2), body background colour pale cream. A broken line comprising 15–17 small, dark-brown spots comprising diffuse clusters of melanophores extending along posterior two-thirds of body; spots located within pocket of scales 11–26 in lateral series. Horizontal septum along posterior two-thirds of body with dark brown pigment forming thin dark brown line deep to broken line. Vertical septum posterior to anal fin with dark brown pigment forming thin dark brown line along ventral midline. Side of body and base of caudal fin with irregular scatter of small dark brown melanophores. Posterior edge of scales, excluding those on predorsal surface, rimmed by small dark brown melanophores forming weak reticulate pattern over body surface. Fins hyaline. Dorsal, lateral, and ventral surface of head with irregular scatter of small dark brown melanophores. Upper lip with dense aggregation of small light brown melanophores forming faint brown line.

**Figure 9.**
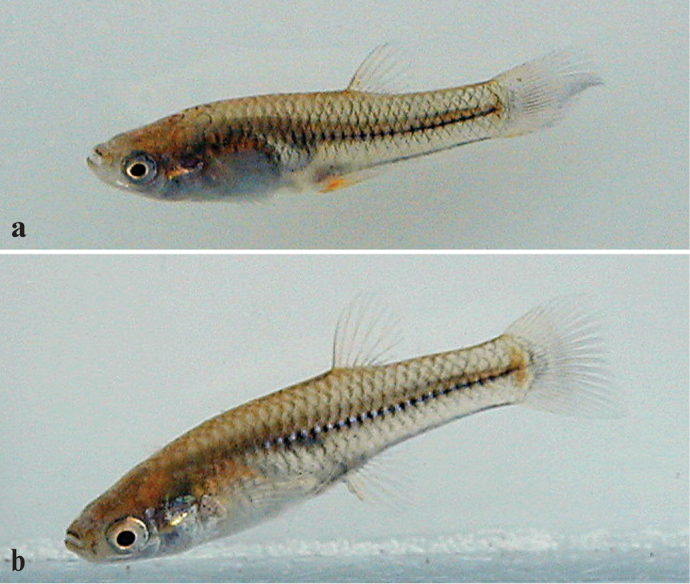
*Poeciliopsis jackschultzi*, Mexico, Sonora, Río Concepción **a** male **b** female. Paratype specimens. Not measured, not identified in field.

In life (Fig. 9), body translucent. Anterodorsal surface of body golden brown; remainder of body faint olive-yellow. Broken line along centre of body comprised of black spots, interspaced by small iridescent white-blue spots. Caudal-fin base with a faint golden-brown oval-shaped marking at centre. Majority of scales with light to dark brown pigment along posterior margin, forming obvious reticulate pattern. Fins of female (Fig. 9b) and paired fins of male hyaline. Dorsal and caudal fin of male with faint orange tint; fin membranes between central caudal-fin rays with faint dark brown or black markings. Base of gonopodium bright orange (Fig. 9a). Dorsal surface of head golden brown. Upper part of opercle with small silver-white marking. Upper lip dark brown. Iris golden-white.

#### Distribution and habitat.

**Figure 10.**
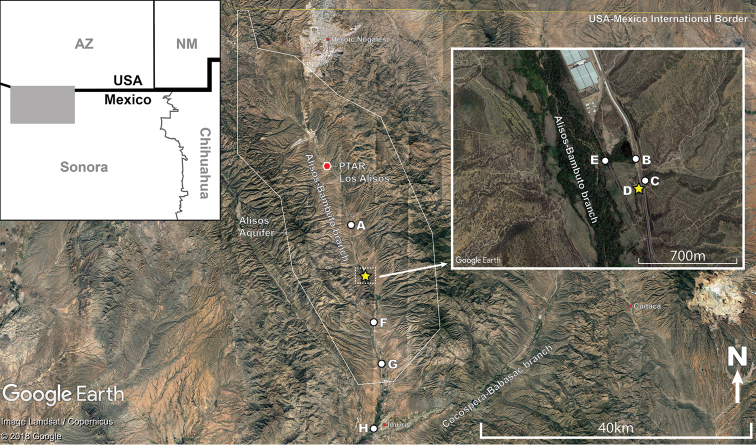
Distribution of sites (A-H) within the Río Concepción from which *Poeciliopsis jackschultzi* has been collected since 1981 (see Table 4). Type locality indicated by yellow star. Grey box in left inset figure indicates area presented in main figure. Right inset shows close up of area highlighted by white box. Extent of the Alisos Aquifer (also known as Rio Alisos Aquifer) is highlighted by a white line. The waste water treatment facility (PTAR Los Alisos) is indicated by a red polygon. The large commercial facility just north of the type locality is currently a Ganfer tomato greenhouse complex.

**Figure 11.**
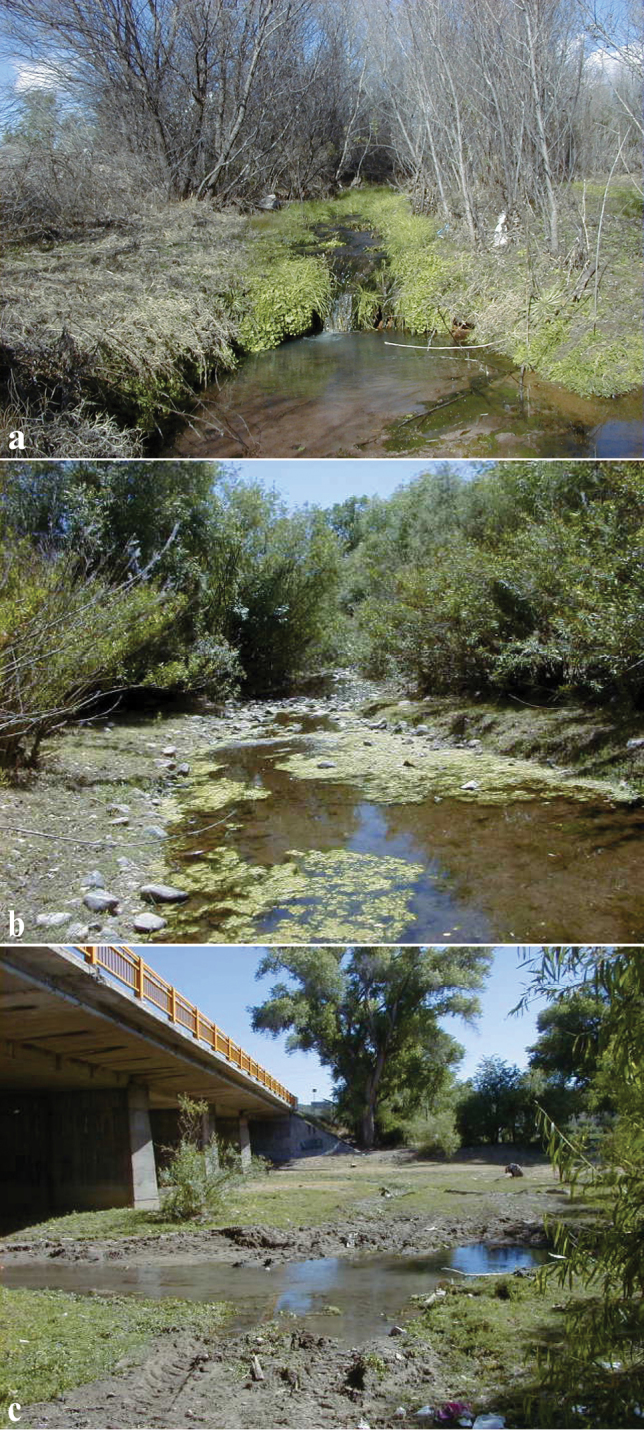
Habitat of *Poeciliopsis jackschultzi***a** La Atascosa, small spring tributary to Alisos-Bambuto branch of the Río Concepción at highway 15 road crossing close to Rancho Semarnap, type locality (site D in Fig. 10, Table 4) **b** pool at Rancho Las Playas, tributary to Alisos-Bambuto branch (site F in Fig. 10, Table 4) **c** Río Concepción at (Cocospera-Babasac branch) at town of Imuris (site H in Fig. 10, Table 4).

Known currently from four sites in the Río Concepción, Sonora State, Mexico (Fig. 10). The type locality (Fig. 11a) is a small intermittent stream that passes under Hwy 15 and the railroad tracks; soon after, it merges into the Alisos-Bambuto branch of the Río Concepción. At the time of collection (January 2001), this site had a narrow ca. 1m wide stream with clear running water, as well as small and shallow pools. Parts of the stream were bordered by trees. The bottom was muddy. Approximately 250m north of the type locality, immediately west of Hwy 15, there is a warm spring (site B in Fig. 10), where *P. jackschultzi* was previously sampled (RCV, pers. obs.). Our 2001 collection, however, yielded only 18 female individuals of *Poeciliopsis*. Nine of these were genotyped for the *Cytb* gene; all shared the same *P. monacha*-derived haplotype (Table 4), and thus were not *P. jackschultzi* (i.e., pending analyses of nuclear markers, they are either *P. monacha-occidentalis* or *P. monacha-jackschultzi* hybrids; see Hybridisation section). Further south, near Rancho Las Playas (site F in Fig. 10), paratype TCWC 20083.01 was collected in an exposed pool of the Alisos-Bambuto branch mainstream. At the time of collection (April 1999), this site had clear running water and maximum depth ~ 60 cm. The nearby spring-fed area that had held water in a previous visit was dry (RCV, pers. obs.). *Poeciliopsis jackschultzi* has also been found in the Babasac-Cocospera branch of the Río Concepción (also intermittent, but larger than the Alisos-Bambuto branch), under the Hwy 15 bridge at the town of Imuris (site H in Figure 10; Fig. 11c). This is the locality of the voucher (MVH99-2a#5) used for molecular phylogenetic analysis by Mateos et al. (2019) and an additional paratype (TCWC 20084.01). This site has a muddy bottom and little aquatic vegetation, except for some floating vegetation. Based on all collections, *P. jackschultzi* seems to prefer marshy or pool sites, with still water or relatively slow current. Adjacent mainstream habitats with deeper water and faster currents favour the native cyprinids *Agosia* and *Gila*.

**Table 3.** Uncorrected P-distances (%) among five members of the *Leptorhaphis* species group and *P. monacha* (outgroup). Nuclear genes (*ENC, Glyt, SH3PX3, Myh6, Rag1, Rh, Xsrc*) above diagonal, mitochondrial genes (*Cytb* and *ND2*) below diagonal.

	***P. jackschultzi***	***P. infans***	***P. lucida***	***P. occidentalis***	***P. prolifica***	***P. monacha***
***P. jackschultzi***	–	0.95	0.67	0.56	0.92	1.35
***P. infans***	7.73	–	0.93	0.95	0.96	1.60
***P. lucida***	5.44	8.70	–	0.54	0.69	1.51
***P. occidentalis***	5.21	8.38	4.02	–	0.78	1.39
***P. prolifica***	6.95	9.38	5.40	5.35	–	1.68
***P. monacha***	16.05	17.12	16.23	16.23	17.28	–

**Table 4.** Abundance of *P. jackschultzi* relative to other *Poeciliopsis* based on non-random sampling (mostly targeted at *P. jackschultzi*). Site letters correspond to labels shown in Figure 10. Type locality and collection (D) bold, underlined. Collections from which paratypes were obtained in bold. Most specimens were identified on the basis of at least the *Pgd* diagnostic allozyme (1981–1989; results from Schenk 1992). An asterisk indicates uncertainty regarding the precise location (i.e., up to ~ 3 km from the depicted coordinates). Similarly, a range of letters (e.g. B–D) implies uncertainty regarding the precise location. Geographic coordinates for the 1999–2001 collections were obtained at time of collection and verified in GoogleEarth. All other geographic coordinates were inferred from field notes, including kilometre marks along Mexico Highway 15.

**Site**	**Latitude**	**Longitude**	**Collection ID**	**Locality Name (Year)**	**Total individuals genotyped**	***P. jackschultzi* (relative abundance)**	***P. monacha-jackschultzi*** ^c^ **(relative abundance)**	**Other *Poeciliopsis***
B-D	–	–	VD81-1	La Atascosa Cienega (1981)	130	21 (16.15%)	32 (24.62%)	77
F-G	30.881	-110.851	VM84-9	La Providencia Cienega (1984)	128	5 (3.91%)	19 (14.84%)	104
G*	30.881	-110.851	VD86-6	La Providencia Cienega (1986)	111	9 (8.11%)	14 (12.61%)	88
E*	30.956	-110.858	VD86-7	La Atascosa Cienega (1986)	92	53 (57.61%)	23 (25.00%)	16
A	31.079	-110.909	VD86-8	La Cieneguita (1986)	134	14 (10.45%)	4 (2.99%)	116
H	30.775	-110.858	VD86-1	Imuris (1986)	145	24 (16.55%)	0 (0.00%)	121
F-G*	30.861	-110.850	VQH89-1	La Providencia mainstream (1989)	83	0 (0.00%)	55 (66.27%)	28
F-G*	–	–	VS94-1	La Providencia Cienega (1994)	30	3 (10.00%)	2 (6.67%)	25
F	30.919	-110.861	MV00-1	Rancho Las Playas (2000)	48	0 (0.00%)	8 (16.67%)	40
**H**	30.775	-110.858	**MV00-11**	Imuris (2000)	150	1 (0.67%)	65 (43.33%)^d^	84
**D**	30.981	-110.872	**MV01-2**	La Atascosa, Rancho Semarnap (2001)	95	5 (5.26%)	12 (12.63%)^d^	78
**F**	30.919	-110.861	**MVH99-1b**	Rancho Las Playas (1999)^a,b^	–	1 (n/a)	–	–
B	30.982	-110.872	MV01-1	La Atascosa (2001)^a,b^	8	0 (n/a)	–	–
H	30.775	-110.858	MVH99-2a	Imuris (1999)^a^	–	1^e^ (n/a)	–	–

^a^ fewer than 10 individuals genotyped; inadequate for meaningful frequency estimation ^b^ based on mitochondrial gene sequence only ^c^ individuals with a heterozygous (fast/slow) genotype at *Pgd* ^d^*Cytb* gene sequenced for three females from MV00-11 and three females from MV01-2. One female (from MV00-11) had identical sequence to *P. monacha-occidentalis* haplotype “d” (GenBank AF047343.1); the other five females had identical sequence to *P. monacha-occidentalis* haplotype “a” (GenBank AF047340.1) ^e^ Specimen used in the molecular phylogeny of Mateos et al. (2019)

Sex ratios in wild caught specimens are typically not significantly different from 1:1 (Table 5).

**Table 5.** Chi-squared test of equal sex-ratios in *Poeciliopsis jackschultzi* from the Río Concepción system (data from Schenk 1992). Sample IDs correspond to “Locality name (year)” in Table 4.

**Sample**	**females**	**males**	**χ^2^**
Río Imuris (1986)	18	17	0.02
La Providencia Cienega (1986)	6	4	2.0
La Providencia mainstream (1989)	0	0	na
La Atascosa (1986)	39	10	17.5*
La Cieneguita (1986)	9	10	0.05

* significant at p = 0.05.

#### Etymology.

Named in honour of R. Jack Schultz, a pioneer of studies on hybridisation and all-female reproduction in *Poeciliopsis*. A noun in the genitive.

#### Genetic distances.

Uncorrected P-distances between *P. jackschultzi* and other members of the *Leptorhaphis* species group (Table 3) range from 5.2–7.7% for a 2147 bp fragment of the mitochondrial genome (concatenated *Cytb* and *ND2*) and 0.5–0.9% for a 6173 bp fragment of the nuclear genome (concatenated *ENC, Glyt, SH3PX3, Myh6, Rag1, Rh, Xsrc*). Uncorrected P-distances for the same fragments of mitochondrial and nuclear DNA were much greater between *P. jackschultzi* and *P. monacha* (16.0% for mitochondrial DNA and 1.3% for nuclear DNA; Table 3).

#### Comparisons.

*Poeciliopsis jackschultzi* differs from all other members of the *Leptorhaphis* species group (viz. *P. infans*, *P. lucida*, *P. occidentalis* s. l., and *P. prolifica*) and all other members of *Poeciliopsis* (excluding *P. monacha*) by having type 3 gill rakers along the anterior edge of ceratobranchials 2–4 (*vs.* type 1b gill rakers along the anterior edge of ceratobranchials 2–4; Fig. 7b). The number of sclerotic bones in the scleral cartilage also differs between the members of the *Leptorhaphis* species group (see Fig. 4) and *P. jackschultzi* can be distinguished from *P. infans*, *P. lucida*, and *P. prolifica*by the absence (*vs.* presence) of the anterior sclerotic, and from *P. occidentalis* s. l. by the presence (*vs.* absence) of the posterior sclerotic. *Poeciliopsis jackschultzi* can be further distinguished from *P. infans*, *P. occidentalis* s. l., and *P. prolifica* by having weakly trifid teeth in the inner row of the dentary and premaxilla (*vs.* conical teeth); from *P. occidentalis* s. l. by the absence (*vs.* presence) of a black spot at the base of the anterior part of the dorsal fin; and from *P. prolifica* by having 6 or 7 pores in the preopercular portion of the preoperculo-mandibular canal (*vs.* canal an open trough, without pores), posterior two-thirds of body with a broken (*vs.* solid) horizontal line along center, and by the absence (*vs.* presence) of two dark brown or black markings on the ventral surface of the head below the preorbit region.

Although distinct, *P. jackschultzi* shares some characteristics with *P. monacha*, a sexually reproducing species presently distributed > 400 km to the south in the Ríos Mayo, Fuerte and Sinaloa. The new species produces hybrids with hemiclonal *monacha* genomes derived from hybridogenetic *P. monacha-occidentalis* females (see Hybridisation section); therefore, introgression of *monacha* characteristics is possible. Nonetheless, *P. jackschultzi* clearly differs from *P. monacha* by having only 7–10 weakly tricuspid teeth arranged in a single row on the lingual surface of the premaxilla and dentary (*vs.* 50+ weakly tricuspid teeth arranged as a dense patch on lingual surface of premaxilla and dentary), the absence (*vs.* presence) of ceratobranchial 4 teeth, and the presence (*vs.* absence) of a retrorse hook at the tip of the gonopodium.

*Poeciliopsis jackschultzi* males do not appear to exhibit the black nuptial colouration exhibited by males of *P. monacha* and certain members of the *Leptorhaphis* group (viz. *P. lucida* and *P. occidentalis* s. l.) (Miller 1960). However, *P. monacha*, *P. lucida* and *P. occidentalis* s. l. males rapidly “turn off” these nuptial displays when subjugated by behaviourally dominant males or if captured in nets (Vrijenhoek and Schultz 1974). The nuptial displays of these species are expressed clearly in aquaria, but we have not observed similar nuptial pigmentation in laboratory-reared *P. jackschultzi* males.

*Poecilopsis jackschultzi* co-inhabits the Río Concepción basin with *P. occidentalis* s. l. and the hybrids *P. monacha*-*jackschultzi* and *P. monacha-occidentalis*. The presence of a black spot at base of the anterior part of the dorsal fin is the most reliable feature distinguishing *P. occidentalis* s. l. from *P. jackschultzi* in the field. We are currently unaware of reliable external morphological character(s) that could serve to distinguish *P. jackschultzi* from females of the two co-occurring hybrid forms of *Poeciliopsis* within the Río Concepción.

#### Remarks.

The type series of *P. jackschultzi* comprises individuals collected from the Río Concepción and subsequently maintained in an aquarium for a short period of time. After death, specimens of the type series were maintained in formalin for several years prior to transfer to alcohol, which resulted in decalcification of the skeleton. Though we managed to successfully clear and double stain a female specimen of *P. jackschultzi* (TCWC 20082.01, 23.0 mm SL) for bone and cartilage investigation, our original attempt to clear and double stain a single male individual (TCWC 20082.01, 19.0 mm SL) was not successful: the bone did not stain with alizarin red S. Our attempts to CT scan the holotype and a single female paratype (TCWC 20082.02, 26.7 mm SL) were also unsuccessful, again likely due to decalcification.

Our description of the skeletal elements of the gonopodium reported herein is based solely on the examination of the poorly stained male paratype (TCWC 20082.01; Fig. 8), viewed with the aid of transmitted light. The retrorse hook is present in this individual and another paratype male (TCWC 20084.01, 19.0 mm SL), but not in another immature paratype male (TCWC 20083.01) that was single stained or the holotype, the tip of the gonopodium in which appears to have been damaged. Miller (1960) considered the retrorse hook to be diagnostic for his *Leptorhaphis* species group and we can confirm, based on the material that we have examined, that this character is present in males of all five members of the group, including *P. prolifica*, a species in which the gonopodium has been reported to be “unmodified at the tip” (Miller 1960: 6).

The state of the available female specimens of *P. jackschultzi* precluded adequate assessment of their genital area pigmentation patterns. All other members of the *Leptorhaphis* species group are characterised by a “pre-anal chevron”, and sparse pigmentation in the genital pit. In contrast, *P. monacha* and *P. viriosa* lack the pre-anal chevron and have much more pigmentation in the genital pit (see drawings in Lima et al. 1996; Vrijenhoek and Schultz 1974).

## Discussion

### Gill rakers

Our examination of cleared and double stained material of *Poeciliopsis* (listed below) has revealed an unexpected diversity in the morphology of the gill rakers. To facilitate discussion, we use numbers to refer to the different types (see materials and methods). The majority of the species of *Poeciliopsis* we have examined exhibit three different types of gill rakers, including type 1a (the largest of the different types, restricted to the anterior edge of the first gill arch; Fig. 6b), type 1b (similar in shape to type 1a but smaller, typically found from the posterior edge of the first gill arch to the anterior edge of the fourth; Fig. 6b, 7), and type 2 (a dorso-ventrally compressed gill raker with 4–5 comb-like projections dorsally; Fig. 6b, 7). In addition to type 1 and type 2 gill rakers, *P. jackschultzi* and *P. monacha* also exhibit type 3 gill rakers along the anterior edge of arches 2–4. Type 3 gill rakers not only exhibit a characteristic trifid shape (Fig. 7a, c), with a central shaft and a pair of lateral process, but also support a variable number of minute conical teeth, which are confined to the base of the central shaft and the lateral processes. In *P. jackschultzi*, the number of teeth associated with each type 3 gill raker is low (ranging from 4–6), whereas in *P. monacha* the number of teeth associated with each type 3 raker is higher (ranging from 10–14). Gill raker teeth were not observed in association with any of the other gill raker types (type 1a, 1b, and 2) present in the material of *Poeciliopsis* that we examined, but are typically found in association with gill rakers throughout the branchial arches of actinopterygian fishes (e.g., see Nelson 1969) and are present in all other members of the Poeciliinae that we examined, including members of *Alfaro*, *Brachyrhaphis*, *Gambusia*, *Heterandria*, *Neoheterandria*, *Phallichthys*, *Poecilia*, and *Priapichthys* (see comparative material). Interestingly, in these latter poeciliine taxa, gill raker teeth were only observed in association with those gill rakers (all type 1b) located along the anterior edge of arches 2–4, mirroring the distribution in *P. jackschultzi* and *P. monacha*. As gill raker teeth are typically found in association with gill rakers located throughout the entire branchial basket in non-poeciliid cyprinodontiforms (and other teleosts), the restricted distribution of gill raker teeth to those gill rakers located along the anterior edge of arches 2–4 in poeciliids is an interesting pattern and, if shown to be present in other members of this group, could represent an additional synapomorphy in support of Poeciliinae, or a more inclusive group. At the level of *Poeciliopsis*, the presence of gill raker teeth on type 3 gill rakers in *P. jackschultzi* and *P. monacha* is most logically interpreted as a symplesiomorphy but further investigation of the gill rakers in *Poeciliopsis*, including observations on other members of the “predominantly Northern” clade of the subgenus Poeciliopsis (viz. *P. balsas* and *P. viriosa*) will be needed to better understand the distribution of this character within the genus.

The majority of the other poeciliids that we examined exhibited only type 1a gill rakers (on the anterior edge of the first arch) and 1b (on the posterior edge of the first gill arch to the anterior edge of the fifth). In addition to type 1a and 1b gill rakers, members of *Neoheterandria*, *Phallichthys* and *Poecilia* also exhibited type 2 gill rakers on the posterior edge of the fourth gill arch (ceratobranchial 4) and the anterior edge of the fifth (ceratobranchial 5), mirroring the condition in *Poeciliopsis*. As in *Poeciliopsis* (Fig. 7), in these aforementioned taxa the posterior edge of ceratobranchial 4 is expanded into a plate-like shelf to accommodate the comb-like type 2 gill rakers. Though comb-like gill rakers are known from other groups of teleosts (e.g., Cypriniformes; Conway 2011: fig. 31), to the best of our knowledge comb-like gill rakers (herein referred to as type 2 gill rakers) have not been reported in recent morphological investigations of poeciliids (e.g., Ghedotti 2000; Lucinda and Reis 2005) or cyprinodontiform fishes more generally (e.g., Parenti 1981; Costa 1998; although see Whitehead [1962] for description [p.119] and illustration [fig. 10] of “tooth-like” gill rakers in *Aplocheilus panchax* and description [p.119] of “tree-like and branched” gill rakers in *Aphanius dispar*). Based on our limited observations, the presence of type 2 gill rakers in poeciliids appears to be correlated with a widening of the bones supporting the pharyngeal jaws and also in the arrangement of the teeth into regular rows on these bones (e.g., Costa 1991, Ghedotti 2000, Lucinda and Reis 2005). These latter characters were interpreted as uniquely derived and unreversed synapomorphies of the supertribe Poeciliini by Lucinda and Reis (2005) and type 2 gill rakers may represent further evidence in support of this group or a more inclusive group. We note here that similar modifications of the bones supporting the pharyngeal jaws have been reported for *Pantanodon* by Whitehead (1962) and Bragança et al. (2018) but without associated modification of the gill rakers or expansion of ceratobranchial 4. A detailed survey of gill raker morphology across Poeciliinae was beyond the scope of this study but may be justified given the diversity that we have uncovered in this character complex based on the examination of only a relatively small number of taxa.

### Hybridisation

Previous allozyme studies clearly revealed the occurrence of hybrids between *P. jackschultzi* and *P. monacha-occidentalis*, the locally occurring all-female hybridogenetic fish (Schenk 1992). The hybridogens are ‘hemiclonal’ transmitting only a maternally inherited, non-recombinant, *monacha* (M) genome to progeny (Schultz 1961, 1969; Vrijenhoek 1977). Their paternally derived *occidentalis* (O) genome is replaced in each generation by matings with *P. occidentalis* males. Consequently, fertilisation of *P. monacha-occidentalis* eggs by sperm from *P. jackschultzi* (J) males produces *P. monacha-jackschultzi* (MJ) hybrids. The MJ hybrids were identified by their heterozygous (fast and slow) genotype at the *Pgd* locus. All of the MJ hybrids identified in our 1999–2001 samples were females (*N* = 85; Table 4), but Schenk (1992) reported the occurrence of two MJ males among ~ 130 MJ females in the 1981–1989 samples. We maintained several MJ hybrid females in the laboratory, some of which produced offspring (also females). Mitochondrial Cytochrome *b* sequences from six of the wild-caught MJ females (three from MV00-11 and three from MV01-2) were identical to the *P. monacha*-derived *Cytb* sequences previously identified by Sanjur (1998) as *P. monacha-occidentalis* haplotypes “a” and “d” from the Rio Concepción (GenBank Acc. No. AF047343 and AF047344), verifying their hybrid status. The rare occurrence of males among hybridogenetic *Poeciliopsis* is not unprecedented (e.g., Schultz 1966, 1967 reported rare *P. monacha*-*latidens* males, which were sterile). Further work is needed to verify the reproduction mode of the MJ hybrids. Should such hybrids be sexual (i.e., have normal recombination and fertility), they could serve as a vehicle of introgression of the hemiclonal *monacha*-derived genome into the *P. jackschultzi* gene pool. Similarly, whether hybridisation (and introgression) occurs between *P. jackschultzi* and *P. occidentalis* has not been determined. Further studies involving a large number of nuclear gene markers are warranted.

### Life history

The *Leptorhaphis* species group exhibits a broad range of placentation and maternal provisioning phenotypes, as measured by the matrotrophic index (MI; Reznick et al. 2002). At the lowest end of maternal provisioning is *P. infans* (MI = 0.86). *Poeciliopsis lucida* and *P. occidentalis* have intermediate levels of maternal provisioning (MI = 1.34 and 1.12, respectively). In contrast, *P. prolifica* has high maternal provisioning (MI = 5.4). Investigating the degree of maternal provisioning in *P. jackschultzi* will likely shed light on the evolution of this complex adaptation within the *Leptorhaphis* species group.

### Conservation status

Several features of *P. jackschultzi* indicate that its conservation status is of concern. First, it has a highly restricted distribution. This is a microendemic species, known from only a handful of sites in the Río Concepción (Fig. 10). During the past 50 years, numerous specimens of *Poeciliopsis* have been collected at numerous other localities in the Río Concepción and neighbouring drainage systems in Arizona and Sonora (Moore et al. 1970; Vrijenhoek et al. 1985), but *P. jackschultzi* was not encountered at any of these localities (RCV pers. obs.). Based on Maderey-R and Torres-Ruata (1990), the Río Concepción is approximately 1000 km in length. Assuming that *P. jackschultzi* is found continuously between the northernmost locality (La Cieneguita; A in Figure 10) and the southernmost locality (at the town of Imuris; H in Figure 10) from which the holotype and members of the paratype series were collected, this species may occupy only an approximately 30km stretch (i.e. 3%) of the Río Concepción. Within this narrow stretch, *P. jackschultzi* has only been collected from marshy, shallow, and relatively still-water habitats, which are patchily distributed throughout the basin (RCV, MM pers. obs.). Secondly, the local abundance of *P. jackschultzi* is low compared to *P. occidentalis* and the hybrids within each locality (below 17% at all sites except for La Atascosa in 1986; Table 4). Collections made after 1984 generally targeted individuals of *P. jackschultzi* and therefore their relative frequency in nature may be lower. Sadly, expansion of Federal Hwy 15 after 1986, nearly obliterated the primary habitat of *P. jackschultzi* at La Atascosa (RCV pers. obs.). The core distribution of *P. jackschultzi* appeared to be mostly spring-fed pools and marshy areas (cienegas) adjacent to the mainstream of Arroyo Alisos-Bambuto branch of the Río Concepción (Figs 10, 11). Although CONAGUA (2018) reports an overall positive recharge *vs.* extraction balance of the Alisos aquifer (Fig. 10), the human demand for water in this desert region continues to increase. From 1990 to 2012, water from the Alisos aquifer was extracted and exported to the city of Nogales. Residual water was transferred to the neighbouring drainage (Rio Santa Cruz) for treatment and release on the US side. Therefore, during this recent ca. 20-year period, recharge of the Alisos aquifer depended solely on precipitation. The Alisos Wastewater Treatment Plant (PTAR Los Alisos; Figure 10), which initiated operation in 2012, releases treated water into the Alisos-Bambuto branch, which is expected to increase water quantity in the mainstream and the aquifer. Nonetheless, pollutant loads of the discharge have been reported above the maximum permissible limits (Meranza-Castillón et al. 2017). An additional factor that may exert further pressure on *P. jackschultzi* is the presence of several exotic species of fishes throughout the Río Concepción, including the mosquitofish *Gambusia affinis* and green sunfish *Lepomis cyanellus* (MM pers. obs.; Hendrickson and Juárez-Romero 1990).

The present status of *P. jackschultzi* and its habitats are unknown, as surveys of this species and its known sites have not been undertaken since 2001. We recommend that future surveys include seining where feasible, as well as the use of minnow traps in the marshy areas where seining is not effective.

### Comparative material

***Alfaro***

***A. cultratus*. **TCWC 3870.16, 2 (C&S); Costa Rica, Heredia, Sarapiqui River drainage, 1 May 1984.

***Brachyrhaphis***

***B. parismina*.**TCWC 3873.10, 2 (C&S); Costa Rica, Heredia, Sarapiqui River drainage, 4 May 1984.

***Gambusia***

***G. affinis*. **TCWC 20085.01, 6 (C&S); USA, Texas, Washington Co., 15 May 2012.

***Heterandria***

***H. formosa*.**TCWC 320.18, 4 (C&S); USA, Florida, Levy Co., 7 June 1975.

***Neoheterandria***

***N. umbratilis*. **TCWC 6264.18, 2 (C&S); Costa Rica, Heredia, Sarapiqui River drainage, 28 April 1985.

***Phallichthys***

***P. amates*.**TCWC 6264.17, 2 (C&S); Costa Rica, Heredia, Sarapiqui River drainage, 28 April 1985.

***Poecilia***

***P. latipinna*. **TCWC 20086.01, 2 (C&S); USA, Texas, Galveston Island, December 2011.

***Poeciliopsis*** (All Mexico unless otherwise noted).

***P. catemaco*.**TCWC 775.07, 3 (C&S); Veracruz, Rio Papaloapan Drainage, Rio Grande (Lago Catemaco outlet), 21 August 1964.

***P. gracilis*. **TCWC 1844.05, 5 (C&S); Chiapas, 2 miles south east of Huixtla, 12 June 1966. – TCWC 6233.05, 8 (C&S); Oaxaca, Los Minas near Zanatepec, 30 May 1983.

***P. fasciata*. **TCWC 3197.08, 3 (C&S); Oaxaca, Las Mina, HWY 190, ca. 3 miles north of Tapanatepec, 31 December 1981.

***Leptorhaphis* species group. *P. infans*.**UMMZ 172172, 30 of 280, 8.0–68.0 mm SL; Michoacan, Rio Lerma Drainage, Lago de Camecuaro, ca. 9 miles south east of Zamora, 9 March 1955. – UMMZ 173631, 36, 25.0–36.0 mm SL; Michoacan, Rio Lerma Drainage, canal at Tarecuato, south west of Zamora, 16 April 1939. – UMMZ 188806, 17 of 124, 17.0–46.0 mm SL; Guanajuato, roadside ditch, 7 miles east of Salamanca along highway between Salamanca and Celaya, 17.0–46.0 mm SL. – UMMZ 189041, 30 of 280 (4 C&S), 20.0–38.0 mm SL; Michoacan, Rio Lerma Drainage, Lago de Cuitzeo at highway 43, 28 March 1968. ***P. lucida*.**UMMZ 178297, 22, 10.0–39.0 mm SL; Sinaloa, Rio de Mocorito just above El Alamo, ca. 24 miles east of Guamuchil, 21 February 1957. – UMMZ 184874, 30 of 76 (4 C&S), 14.0–34.0 mm SL; Sinaloa, tributary of the Rio Mocorito, at road crossing 0.9 miles north of San Benito, 24 March 1959. – UMMZ 184897, 13, 15.0–27.0 mm SL; Sinaloa, tributary of the Rio Mocorito, ca. 2.5 miles West of El Alamo, 22 February 1957. – UMMZ 188915, 30 of 142, 12.0–20.0 mm SL; Sinaloa, stream 1.1 miles south of El Rincon de Carrizalejo, 25 February 1969. ***P. occidentalis*.**UMMZ 162670, 30 (of 115), 17.0–35.0 mm SL; Sonora, Gila River Drainage, Rio Santa Cruz, 4 miles south of US (Arizona) border, 19 April 1950. – UMMZ 202393, 41 (4 C&S), 15.0–42.0 mm SL; Sonora, stream at Rancho la Brisca, 16 km north east of Cucurpe, 5 June 1978. – UMMZ 211632, 29 (of 169), 9.0–31.0 mm SL; Sonora, Rio Yaqui drainage, Arroyo El Fresno, south west of Cabullonas, 18 July 1978. ***P. prolifica*.**UMMZ 172267, 30 or 456 (4 C&S), 8.0–36.0 mm SL; Sonora, Rio Culiacan Drainage, Arroyo Sonolona, 18.5 miles east of Culiacan, 2 April 1955. – UMMZ 173677, 17 of 114, 4.0–35 mm SL; Sinaloa, Rio Culiacan Drainage, Rio Tamazula, 6 km east of Culiacan.

***P. monacha*. **UMMZ 178246, 30 of 379 (4 C&S), 12.0–27.0 mm SL; Sonora, Rio del Fuerte Drainage, Arroyo San Benito, ca. 1.5 miles east south east of Rancho Guirocoba, 16 February 1957.

***P. pleurospilus*.**TCWC 16342.04, 5 (C&S); El Salvador, Santa Ana, Laguna Metapan, 6 June 2011.

***P. scarlii*.**UMMZ 178422, 30 of 127 (4 C&S), 10.0–40.0 mm SL; Guerrero/Michoacan, Rio Balsas at Zacatula, 18 March 1957. – UMMZ 178506, 18 (of 62), 18.0–49.0 mm SL; Guerrero, Laguna Tres Palos at northwest end near Acapulco Airport, 23 March 1957.

***Priapichthys***

***P. annectens*.**TCWC 6268.10, 2 (C&S); Costa Rica, Heredia, Sarapiqui River drainage, 25 April 1985.

## Supplementary Material

XML Treatment for Poeciliopsis jackschultzi

## References

[B1] Avise JC (2015) Evolutionary perspectives on clonal reproduction in vertebrate animals. Proceedings of the National Academy of Sciences USA 112: 8867–8873. 10.1073/pnas.1501820112 PMC4517198 26195735

[B2] Bragança PH Amorim PF Costa WJEM (2018) Pantanodontidae (Teleostei, Cyprinodontiformes), the sister group to all other cyprinodontoid killifishes as inferred by molecular data. Zoosystematics and Evolution 94: 1–137. 10.3897/zse.94.22173

[B3] Bussing WA (2008) A new species of poeciliid fish, *Poeciliopsis santaelena*, from Peninsula Santa Elena, Area de Conservación Guanacaste, Costa Rica. Revista de Biología Tropical 56: 829–838. http://www.scielo.sa.cr/scielo.php?script=sci_arttext&pid=S0034-77442008000200031&lng=en&nrm=iso 19256446 10.15517/rbt.v56i2.5626

[B4] CONAGUA (2018) CONAGUA, Clave 2613. http://sina.conagua.gob.mx/sina/tema.php?tema=acuiferos&n=regional [Accessed 11 May 2019]

[B5] Conway KW (2011) Osteology of the South Asian Genus *Psilorhynchus* McClelland, 1839 (Teleostei: Ostariophysi: Psilorhynchidae), with investigation of its phylogenetic relationships within the order Cypriniformes. Zoological Journal of the Linnean Society 163: 50–154. 10.1111/j.1096-3642.2011.00698.x

[B6] Costa WJEM (1991) Description d’une nouvelle espècedu genre *Pamphorichthys* (Cyprinodontiformes: Poeciliidae) du bassin de l’Araguaia, Brésil. Revue Française d’Aquariologie Herpétologie 18: 39–42.

[B7] Costa WJEM (1998) Phylogeny and classification of the Cyprinodontiformes (Euteleostei: Atherinomorpha): A reappraisal. In: Malabarba LR Reis RE Vari RP Lucena ZM Lucena CAS (Eds) Phylogeny and Classification of Neotropical Fishes.Edipucrs, Porto Alegre, 537–560.

[B8] Ghedotti MJ (2000) Phylogenetic analysis and taxonomy of the poecilioid fishes (Teleostei, Cyprinodontiformes). Zoological Journal of the Linnean Society 130: 1–53. 10.1111/j.1096-3642.2000.tb02194.x

[B9] Hendrickson DA Juárez-Romero L (1990) Los peces de la cuenca del Río de la Concepción, Sonora, México, y el estatus del charalito sonorense, *Gila ditaenia*, una especie en amenaza de extinción. Southwestern Naturalist 35: 177–187. 10.2307/3671540

[B10] Lima NRW Koback CJ Vrijenhoek RC (1996) Evolution of sexual mimicry in sperm-dependent clonal forms of *Poeciliopsis* (Pisces: Poeciliidae). Journal of Evolutionary Biology 9: 185–203. 10.1046/j.1420-9101.1996.9020185.x

[B11] Lucinda PHF Reis RE (2005) Systematics of the subfamily Poeciliinae Bonaparte (Cyprinodontiformes: Poeciliidae), with an emphasis on the tribe Cnesterodontini Hubbs. Neotropical Ichthyology 3: 1–60. 10.1590/S1679-62252005000100001

[B12] Maderey-R LE Torres-Ruata C (1990) Hidrografía, escala 1:4000000. In: Hidrografía e hidrometría. Tomo II, Sección IV, 6.1. Atlas Nacional de México (1990–1992). Instituto de Geografía, UNAM, México.

[B13] Mateos M Vrijenhoek RC (2004) Independent origins of allotriploidy in the fish genus *Poeciliopsis*. Journal of Heredity 96: 32–39. 10.1093/jhered/esi010 15598712

[B14] Mateos M Sanjur OI Vrijenhoek RC (2002) Historical biogeography of the livebearing fish genus *Poeciliopsis* (Poeciliidae: Cyprinodontiformes). Evolution 56: 972–984. 10.1111/j.0014-3820.2002.tb01409.x 12093032

[B15] Mateos M Domínguez‐Domínguez O Varela‐Romero A (2019) A multilocus phylogeny of the fish genus *Poeciliopsis*: Solving taxonomic uncertainties and preliminary evidence of reticulation. Ecology and Evolution 9: 1845–1857. 10.1002/ece3.4874 30847076 PMC6392363

[B16] Meranza-Castillón V Ruiz-Hernandez S Ortiz-Navar B Gutiérrez-Gutiérrez R (2017) Impacto de la descarga de agua tratada en la cuenca los Alisos. Revista de Ciencias Ambientales y Recursos Naturales 3: 18–28. https://www.itson.mx/publicaciones/rlrn/Documents/v3-n1-8-impacto-de-la-descarga-de-aguas-residuales.pdf

[B17] Miller RR (1960) Four new species of viviparous fishes, genus *Poeciliopsis*, from northwestern Mexico. Occasional Papers of the Museum of Zoology University of Michigan 619: 1–11. http://hdl.handle.net/2027.42/57108

[B18] Miller RR (1975) Five new species of Mexican poeciliid fishes of the genera *Poecilia*, *Gambusia*, and *Poeciliopsis*. Occasional Papers of the Museum of Zoology University of Michigan 672: 1–44. http://hdl.handle.net/2027.42/57108

[B19] Moore WS Miller RR Schultz RJ (1970) Distribution, adaptation and probable origin of an all-female form of *Poeciliopsis* (Pisces: Poeciliidae) in northwestern Mexico. Evolution 24: 789–795. 10.1111/j.1558-5646.1970.tb01813.x 28564949

[B20] Nelson GJ (1969) Gill arches and the phylogeny of fishes, with notes on the classification of vertebrates. Bulletin of the American Museum of Natural History 141: 475–552. http://hdl.handle.net/2246/1162

[B21] Parenti LR (1981) A phylogenetic and biogeographic analysis of cyprinodontiform fishes (Teleostei, Atherinomorpha). Bulletin of the American Museum of Natural History 168: 335–557. http://hdl.handle.net/2246/438

[B22] Regan CT (1913) A revision of the cyprinodont fishes of the subfamily Poeciliinae. Proceedings of the Zoological Society of London 1913: 977–1018. 10.1111/j.1096-3642.1913.tb02002.x

[B23] Reznick DN Mateos M Springer MS (2002) Independent origins and rapid evolution of the placenta in the fish genus *Poeciliopsis*. Science 298: 1018–1020. 10.1126/science.1076018 12411703

[B24] Rosen DE Bailey RM (1963) The poeciliid fishes (Cyprinodontiformes): their structure, zoogeography, and systematics. Bulletin of the American Museum of Natural History 126: 1–176. http://hdl.handle.net/2246/1123

[B25] Sanjur OI (1998) Molecular Systematics and Evolution of Fish in the Genus *Poeciliopsis* (Cyprinodontiformes: Poeciliidae). PhD Thesis, University of New Jersey, New Brunswick.

[B26] Schenk M (1992) A new sexual species of *Poeciliopsis* with clonal origins. MSc Thesis, Rutgers University, New Brunswick.

[B28] Schultz RJ (1966) Hybridization experiments with an all-female fish of the genus *Poeciliopsis*. Biological Bulletin 130: 415–429. 10.2307/1539747

[B27] Schultz RJ (1967) Gynogenesis and triploidy in the viviparous fish *Poeciliopsis*. Science 157: 1564–1567. 10.1126/science.157.3796.1564 6038168

[B29] Schultz RJ (1969) Hybridization, unisexuality, and polyploidy in the teleost *Poeciliopsis* (Poeciliidae) and other vertebrates. American Naturalist 103: 605–619. 10.1086/282629

[B30] Swofford DL (2002) PAUP*. Phylogenetic Analysis Using Parsimony (*and Other Methods). Version 4. Sinauer Associates, Sunderland.

[B31] Tarby ML Webb JF (2003) Development of the supraorbital and mandibular lateral line canals in the cichlid, *Archocentrus nigrofasciatus*. Journal of Morphology 254: 44–57. 10.1002/jmor.10045 12420320

[B32] Taylor WR Van Dyke GG (1985) Revised procedure for staining and clearing small fishes and other vertebrates for bone and cartilage study. Cybium 9: 107–119. http://sfi-cybium.fr/sites/default/files/pdfs-cybium/01-Taylor%5B92%5D107-119.pdf

[B33] Vrijenhoek RC Douglas ME Meffe GK (1985) Conservation genetics of endangered fish populations in Arizona. Science 229: 400–402. 10.1126/science.229.4711.400 17795900

[B34] Vrijenhoek RC (1993) The origin and evolution of clones versus the maintenance of sex in *Poeciliopsis*. Journal of Heredity 84: 388–395. 10.1093/oxfordjournals.jhered.a111359

[B35] Vrijenhoek RC (1994) Unisexual fish: Models for studying ecology and evolution. Annual Review of Ecology and Systematics 25: 71–96. 10.1146/annurev.es.25.110194.000443

[B36] Vrijenhoek RC Dawley RM Cole CJ Bogart JP (1989) A list of known unisexual vertebrates. In: Dawley R Bogart J (Eds) Evolution and Ecology of Unisexual Vertebrates.Bulletin 466, New York State Museum, Albany, 19–23.

[B37] Vrijenhoek RC Schultz RJ (1974) Evolution of a trihybrid unisexual fish (*Poeciliopsis*, Poeciliidae). Evolution 28: 205–319. 10.1111/j.1558-5646.1974.tb00750.x 28563275

[B38] Webb JF Shirey JE (2003) Postembryonic development of the cranial lateral line canals and neuromasts in zebrafish. Developmental Dynamics 228: 370–385. 10.1002/dvdy.10385 14579376

[B39] Whitehead PJP (1962) The Pantanodontinae, edentulous toothcarps from East Africa. Bulletin of the British Museum of Natural History 9: 105–137. 10.5962/bhl.part.16339

[B40] Woolman AJ (1894) Report on a collection of fishes from the rivers of central and northern Mexico. Bulletin of the U.S. Fish Commission 14: 55–66. https://www.biodiversitylibrary.org/bibliography/3971#/summary

